# Spatial heterogeneity of the microbiota in *Cypripedium franchetii* and Its correlation with organ-specific metabolomes

**DOI:** 10.3389/fpls.2026.1751651

**Published:** 2026-03-25

**Authors:** Ying Tang, Mingyu Dang, Wenwen Xie, Xiaoyu Chen, Erhao Zhang, Zhongbin Wang

**Affiliations:** 1 College of Forestry and Grassland, Xizang Agricultural and Animal Husbandry University, Linzhi, Xizang, China; 2 College of Food Science and Technology, Xizang Agricultural and Animal Husbandry University, Linzhi, Xizang, China; 3 College of Resources and Environment, Xizang Agricultural and Animal Husbandry University, Linzhi, Xizang, China

**Keywords:** correlation, *Cypripedium franchetii*, metabolomes, microbiota, organ-specific

## Abstract

**Introduction:**

*Cypripedium franchetii*, a plant of ornamental and medicinal value, is designated as a Grade II Protected Species in China. The *C. franchetii* population inhabiting the fragile ecosystem at Galongla Pass exhibits unclear patterns in microbial composition across rhizosphere soil, root, stem, and leaf tissues, as well as metabolite distribution and plant tissue-specific microbe-metabolite relationships.

**Methods:**

This study focused on *C. franchetii*, a plant growing in the fragile ecosystem of Galongla Pass in Tibet. We employed an integrated approach combining high-throughput amplicon sequencing (targeting bacterial 16S rRNA genes and fungal ITS regions) and untargeted metabolomics (LC-MS/MS) to systematically analyze the microbial community structure and metabolite distribution in its rhizosphere soil and root, stem, and leaf tissues. We further explored the associations between endophytic microorganisms and metabolites within plant tissues.

**Results:**

The results revealed significant differences in microbial composition across plant compartments: distinct variations were observed between rhizosphere soil and plant tissues, while stem and leaf microbial communities exhibited greater similarity. At the phylum level, Pseudomonadota dominated among bacteria, while Basidiomycota and Ascomycota were the predominant fungal phyla. At the genus level, dominant taxa showed tissue specificity: *Cronobacter* and *Lactobacillus* were dominant bacterial genera in roots, whereas *Acinetobacter* dominated in stems, and *Acinetobacter* and *Agrobacterium* were prominent in leaves. For fungi, *Tulasnella* was the dominant genus in rhizosphere soil and roots, while *Dioszegia* prevailed in stems and leaves. Metabolite analysis indicated significant differences in metabolic profiles among tissues, with stem and leaf metabolite compositions being relatively similar. Correlation analysis further revealed statistically significant correlations between differential microorganisms and differential metabolites in roots and stems, identifying 31 microbial genera significantly correlated with 48 high-abundance metabolites.

**Discussion:**

This study systematically unveils the tissue-specific microecological and metabolic characteristics of *C. franchetii* during its post-flowering nutrient accumulation phase. Key findings include: microbial community assembly involves cooperative mechanisms between core conserved taxa (e.g., *Tulasnella*) and habitat-specific taxa; microbial diversity exhibits a gradient decline from the rhizosphere into plant tissues accompanied by functional group succession; and extensive yet specific potential interaction networks (based on statistical covariation) exist between microorganisms and host metabolites, indicating potential microbial involvement in regulating plant secondary metabolism. These findings not only provide guidance for the conservation of *C. franchetii* (requiring consideration of both core symbiotic and habitat-specific taxa) and constructing synthetic microbial communities during artificial propagation, but also offer a new theoretical basis for the targeted regulation of medicinal active ingredient synthesis through the microbiome.

## 1 Introduction

The Orchidaceae, as the second largest family of flowering plants, represents not only a group of high ornamental value but also an important source of medicinal plants. Most orchid species thrive in specialized habitats characterized by nutrient-poor soils and heterogeneous light conditions, leading to exceptional microbial dependency for their survival and reproduction. Rhizosphere and endophytic microorganisms profoundly influence orchids’ ecological adaptation and drive the formation of bioactive medicinal compounds through mechanisms such as nutrient acquisition, stress alleviation, and secondary metabolite regulation (Gao et al., 2019; Wang et al., 2020; Yang et al., 2020; Chen et al., 2020; Zhang et al., 2020; Li et al., 2022; Xu et al., 2023). This intricate relationship has emerged as a pivotal research focus in orchid biology.

The regulatory mechanisms through which microorganisms influence orchid hosts involve several key processes. Microorganisms directly participate in host nutrient uptake and enhance stress resistance. For instance, rhizosphere microbiomes play a crucial role in the health maintenance and growth of *Dendrobium* species, significantly improving nutrient acquisition efficiency, disease resistance, and stress tolerance (Sarsaiya et al., 2025). During seed germination, orchid seeds depend on specific microorganisms for carbon sources. They hydrolyze fungal trehalose through intrinsic trehalase to obtain glucose while utilizing alternative carbon sources such as amino acids (Zhao et al., 2024). Concerning secondary metabolites, Alkaloids serve as core contributors to the medicinal value of orchids, and their accumulation is closely linked to microbial community structures (Liu et al., 2019). Research indicates significant microenvironmental heterogeneity across plant organs, which drives microbial community differentiation and leads to spatially distinct accumulation patterns of metabolites (Liu et al., 2019; He et al., 2021; Li et al., 2024a; Yu et al., 2024). This provides critical insights for deciphering the formation mechanisms of bioactive compounds in orchids.

*Cypripedium franchetii* is listed as a Category II protected plant in China (State Council of the People’s Republic of China, 2021) and categorized as Vulnerable (VU) on the IUCN Red List of Threatened Species (IUCN, 2023). This species exhibits a distinctive deep sac-shaped labellum in purplish-red hues, conferring significant ornamental value. Medicinally, its roots and rhizomes contain bioactive compounds with therapeutic effects for regulating qi, activating blood circulation, and relieving cough and asthma (Ai, 2021). Notably, Cypripedin—a compound widely distributed across Cypripedium rhizomes—has been pharmacologically confirmed to inhibit lung cancer metastasis effectively (Treesuwan et al., 2018), further highlighting the species’ potential for pharmaceutical development.

Current research on *C. franchetii* remains relatively limited. Wang et al. (2023) first discovered its distribution in Tibet, specifically in the scree slope of the Galongla Pass, an ecosystem characterized by high fragility. The mechanisms underlying microbe-plant interactions within this unique habitat remain unclear. Therefore, this study focuses on *C. franchetii* populations at the Galongla Pass in Tibet. We aim to systematically analyze the microbial diversity and functional profiles in rhizospheric soil and distinct plant tissues (roots, stems, leaves), while conducting an in-depth exploration of the correlations between tissue-specific microbiota structures and metabolites. This work is expected to provide critical theoretical foundations and research bases for future investigations into the regulatory mechanisms of microbes on *C. franchetii*’s ecological adaptation, as well as their influence on the biosynthesis of medicinal active compounds in this species. Additionally, it offers scientific references for the conservation and sustainable utilization of endangered orchid species.

## 2 Materials and methods

### 2.1 Field sampling protocol

In July 2025, 60 *Cypripedium franchetii* plants in the flower withering stage with intact soil were collected at Galongla Pass, Bomi County, Linzhi City, Tibet. Among them, 30 plants were designated for microbiome sequencing: Rhizosphere soil was collected using the shaking method and sieved through a 40-mesh sieve. The plants were rinsed under running water to separate root, stem, and leaf tissues, followed by surface sterilization with 75% ethanol for 30 seconds, immersion in 2% NaClO for 2 minutes, and three rinses with sterile water. The remaining 30 plants were used for metabolome sequencing: After rinsing under running water, roots, stems, and leaves were directly separated and rinsed three times with sterile water. All samples were freeze-dried at -40 °C (SCIENTZ-20F/A) for 45 hours. Post-drying, each sample was aliquoted into three biological replicates and dispatched to Shanghai Majorbio Bio-pharm Technology Co., Ltd. and Shanghai OE Biotech Co., Ltd., respectively. In this study, the samples were designated as follows: MT for rhizosphere soil, MG for root, MJ for stem, and MY for leaf.

### 2.2 High-throughput amplicon sequencing of the 16S rRNA gene and ITS region

DNA extraction and purification, PCR amplification, library construction, and sequencing were outsourced to Shanghai MagiBio Biomedical Technology Co., Ltd. The V1-V3 hypervariable regions of bacterial 16S rRNA genes were amplified using primers 27F (5′-AGAGTTTGATCMTGGCTCAG-3′) and 1492R (5′-GGTTACCTTGTTACGACTT-3′) (Lane, 1991). The partial ITS1 to entire ITS2 regions of fungi were amplified with primers ITS1F (5′-CTTGGTCATTTAGAGGAAGTAA-3′) and ITS4R (5′-TCCTCCGCTTATTGATATGC-3′) (Gardes and Bruns, 1993; White et al., 1990). Sequencing data were deposited in the NCBI database under accession number PRJNA1364301.

All data analyses were conducted on the Majorbio Cloud Platform (https://www.majorbio.com) using the “Microbial Diversity QIIME2 Pipeline Analysis” module. Optimized sequences after quality control and assembly were denoised using DADA2_CCS, resulting in sequences typically referred to as Amplicon Sequence Variants (ASVs). ASVs mapping to Chloroplast and Mitochondrial sequences were removed. To minimize the impact of sequencing depth differences on diversity analyses, data were subsampled to the minimum sequence count per sample. Bacterial and fungal ASVs were taxonomically annotated using the SILVA database (version 138) and UNITE database (version 8.0), respectively. Alpha diversity analysis was performed using Mothur v1.48.3. Principal Coordinates Analysis (PCoA) based on Bray-Curtis distance matrices was conducted using the vegan package in R v3.3.1. Heatmaps depicting species abundance to visualize community composition were generated using the pheatmap v1.0.8 package in R v3.3.1. Linear Discriminant Analysis Effect Size (LEfSe) analysis was performed using the platform’s built-in tool to identify statistically significant biomarker taxa between groups. Functional prediction for bacterial ASVs was performed using PICRUSt2 v2.2.0-b to obtain KEGG pathway abundance information. Fungal ASVs were functionally annotated for guild assignment using FUNGuild v1.0.

### 2.3 Untargeted metabolomic analysis based on liquid chromatography-tandem mass spectrometry

Sample pretreatment, metabolite extraction, LC-MS (SA) full-scan detection, and data preprocessing were outsourced to Shanghai OE Biotech Co., Ltd. Data analysis was performed on the OE Cloud Platform (https://cloud.oebiotech.com). Differential metabolites were screened using the criteria: p-value < 0.05 and FC ≥ 2.0 or FC ≤ 1/2.0. Pathway enrichment analysis of KEGG IDs for differential metabolites was conducted to understand alterations in metabolic pathways among differential samples. Pathways were considered significantly enriched when p-value ≤ 0.05.

### 2.4 Microbial and metabolite correlation analysis

Correlation analysis was conducted between core microbiota (bacterial/fungal genera with ≥1% relative abundance) and the top 50 Level 1 metabolites (confidence level defined by Metabolomics Standards Initiative criteria) across root, stem, and leaf compartments. Additionally, correlations were examined between significantly differential microorganisms at the ASV level in roots versus stems and the top 50 metabolites with the smallest p-values among significantly differential metabolites at Level 1. All correlation analyses were conducted on the OE Cloud Platform.

## 3 Results

### 3.1 Sequencing depth validation and taxonomic unit enumeration

**Figure 1:**
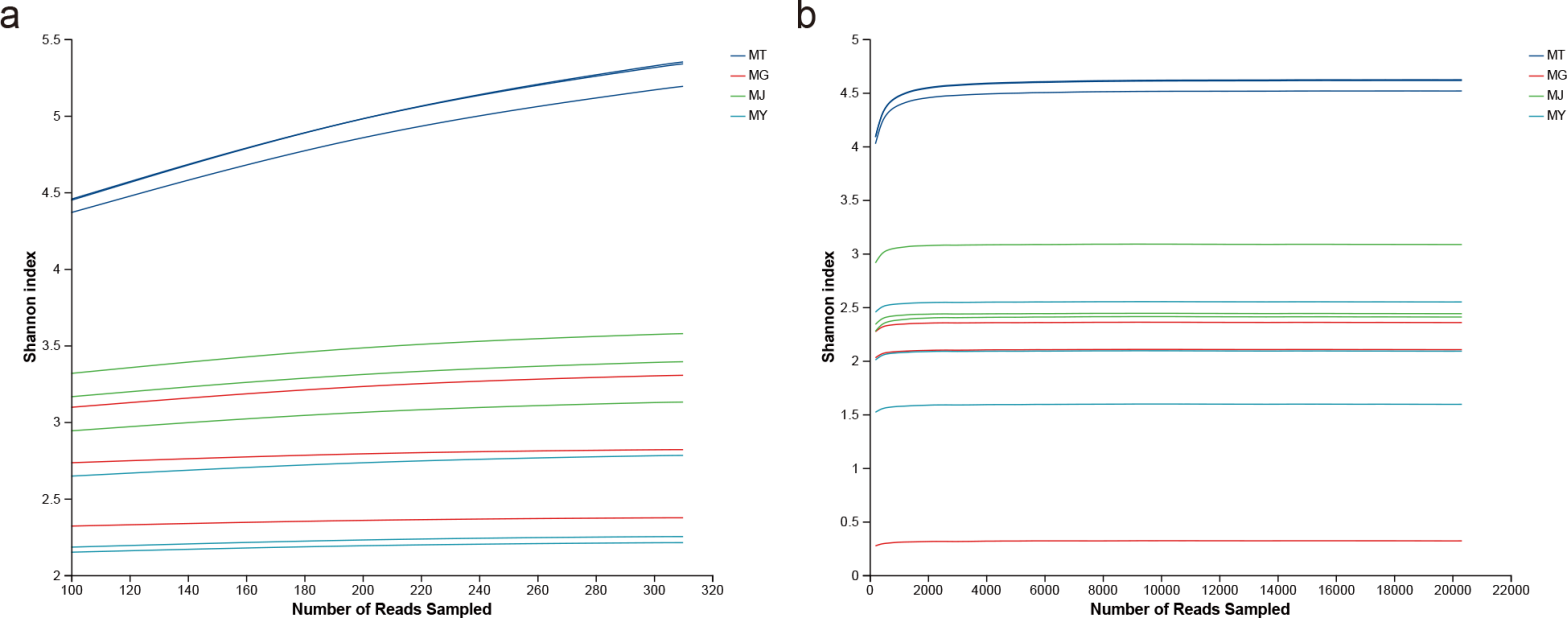
Rarefaction curves of the bacterial **(A)** and fungal **(B)** communities from different compartments of *C. franchetii*: rhizosphere soil (MT) and endophytic tissues of the root (MG), stem (MJ), and leaf (MY).

The dilution curves demonstrate the diversity differences among various samples, with each curve leveling off, indicating that the sequencing depth has essentially covered all species present in the samples. The sequencing data volume was considered sufficient to reflect the species diversity within the samples (**Figure 1**).

A total of 932,471 quality-controlled optimized bacterial sequences were obtained from 12 samples (rhizosphere soil, roots, stems, and leaves) of *C. franchetii*. After denoising, 363,364 high-quality sequences remained. ASVs aligned to Chloroplast and Mitochondrial sequences were removed, and the data were rarefied to 310 sequences per sample based on the minimum sequence count, yielding 994 ASVs. These were annotated to 24 Phyla, 54 Classes, 93 Orders, 143 Families, 251 Genera, and 351 Species. For fungal analysis, 630,530 quality-controlled optimized sequences were obtained. Following denoising, 364,960 high-quality sequences were retained. After excluding ASVs aligned to Chloroplast and Mitochondrial sequences, rarefaction to 20,327 sequences per sample resulted in 1,385 ASVs. Annotation identified 12 Phyla, 35 Classes, 79 Orders, 177 Families, 274 Genera, and 331 Species (**Table 1**).

**Table 1:** Taxonomic composition of bacterial and fungal communities in rhizosphere soil and endophytic tissues (root, stem, leaf) of *C. franchetii*.

Microorganisms	Domain	Kingdom	Phylum	Class	Order	Family	Genus	Species	ASVs
Bacteria	2	2	24	54	93	143	251	351	994
Fungi	1	1	12	35	79	177	274	331	1385

### 3.2 Alpha diversity indices assessment

The bacterial community richness and diversity in rhizosphere soil were significantly higher than those in roots, stems, and leaves. Root, stem, and leaf samples exhibited comparable bacterial richness, but leaf bacterial diversity was significantly lower than that of stems (p < 0.05). The Simpson index of rhizosphere soil reached 0, indicating the absence of dominant bacterial species. The Phylogenetic Diversity values of rhizosphere soil and stems were significantly higher than those of roots and leaves, suggesting greater phylogenetic diversity in these compartments. Similarly, rhizosphere soil demonstrated the highest fungal richness and diversity, significantly exceeding levels in roots, stems, and leaves. Roots exhibited the lowest fungal richness, significantly lower than stems. Fungal communities in roots, stems, and leaves showed higher dominance compared to rhizosphere soil, suggesting potential dominance by limited fungal taxa. The Phylogenetic Diversity value of rhizosphere soil significantly surpassed those of roots, stems, and leaves, indicating greater phylogenetic diversity in its fungal community (**Table 2**).

**Table 2:** Alpha diversity indices of bacterial and fungal communities in rhizosphere soil and endophytic compartments (root, stem, leaf) of *C. franchetii*.

Sample	Bacteria				Fungi			
	Chao	Shannon	Simpson	Phylogenetic diversity	Chao	Shannon	Simpson	Phylogenetic diversity
MT	596.17 ± 116.26a	5.29 ± 0.09a	0.00 ± 0.00c	21.67 ± 0.25a	313.40 ± 24.27a	4.58 ± 0.06a	0.02 ± 0.00	47.25 ± 2.21a
MG	30.17 ± 21.45b	2.83 ± 0.47bc	0.08 ± 0.03b	5.73 ± 1.63b	38.67 ± 3.06c	1.59 ± 1.11b	0.43 ± 0.42	10.23 ± 2.42b
MJ	62.16 ± 11.76b	3.37 ± 0.23b	0.05 ± 0.01b	15.36 ± 7.07a	64.33 ± 14.84b	2.64 ± 0.38b	0.20 ± 0.06	8.78 ± 1.99b
MY	22.33 ± 11.93b	2.41 ± 0.32c	0.12 ± 0.03a	6.31 ± 2.32b	45.33 ± 2.08bc	2.08 ± 0.48b	0.25 ± 0.11	9.54 ± 1.27b

The abbreviations represent the following sample types: MT (rhizosphere soil), MG (root), MJ (stem), and MY (leaf). Different lowercase letters indicate statistically significant differences at p < 0.05.

### 3.3 Community similarity and dissimilarity profiling

**Figure 2:**
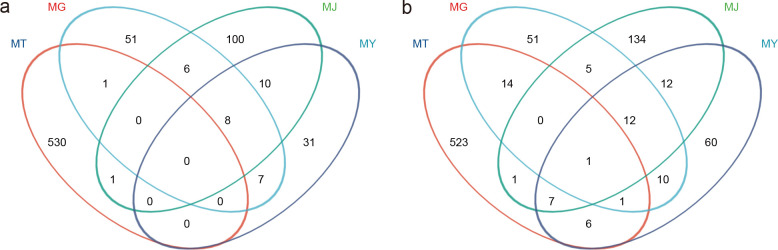
Venn diagrams illustrating the shared and unique bacterial **(A)** and fungal **(B)** taxa at the ASV level across different compartments of *C. franchetii*: rhizosphere soil (MT) and endophytic tissues of the root (MG), stem (MJ), and leaf (MY).

Rhizosphere soil harbored 530 unique bacterial ASVs, while roots, stems, and leaves contained 51, 100, and 31 unique bacterial ASVs, respectively. Eight bacterial ASVs were shared among roots, stems, and leaves. No bacterial ASVs were shared across rhizosphere soil, roots, stems, and leaves (**Figure 2A**). For fungal communities, rhizosphere soil exhibited 523 unique ASVs, with roots, stems, and leaves containing 51, 134, and 60 unique fungal ASVs, respectively. Twelve fungal ASVs were shared among roots, stems, and leaves. A single bacterial ASV was shared across all four compartments (rhizosphere soil, roots, stems, and leaves) (**Figure 2B**).

**Figure 3:**
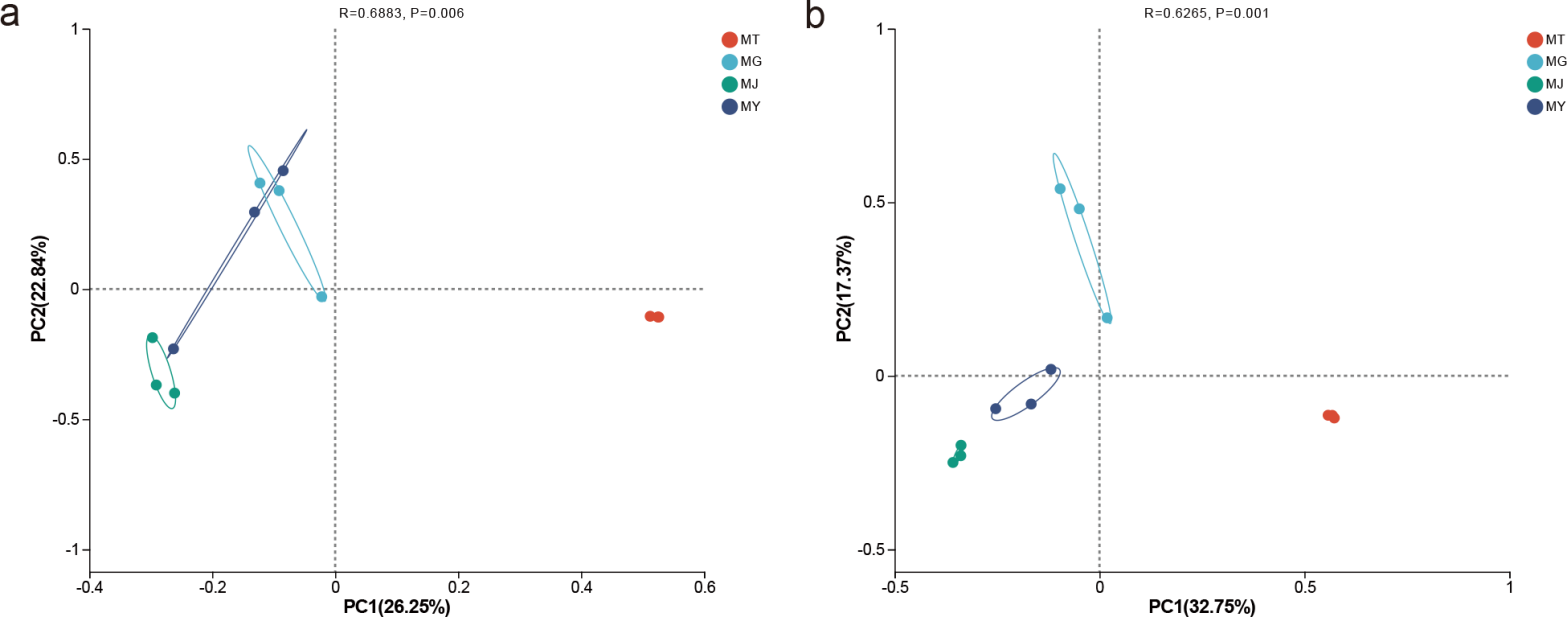
Principal coordinate analysis (PCoA) of the bacterial **(A)** and fungal **(B)** communities based on Bray-Curtis dissimilarity across different compartments of *C. franchetii*: rhizosphere soil (MT) and endophytic tissues of the root (MG), stem (MJ), and leaf (MY).

Based on Bray-Curtis distances, PCoA analysis of ASVs revealed that bacterial community variation was collectively explained by PC1 (26.25%) and PC2 (22.84%) at 49.09%. ANOSIM confirmed highly significant intergroup differences (R = 0.6883, P = 0.006). Results demonstrated that rhizosphere soil bacterial community structure exhibited significant differences from plant tissues, while root and leaf bacterial communities showed structural similarity (**Figure 3A**). For fungal communities, PC1 (32.75%) and PC2 (17.37%) collectively explained 50.12% of community variation, with ANOSIM indicating highly significant intergroup differences (R = 0.6265, P = 0.001). Findings revealed distinct fungal community structures between rhizosphere soil and plant tissues, along with detectable variations among root, stem, and leaf fungal communities (**Figure 3B**).

### 3.4 Microbial community abundance structure

**Figure 4:**
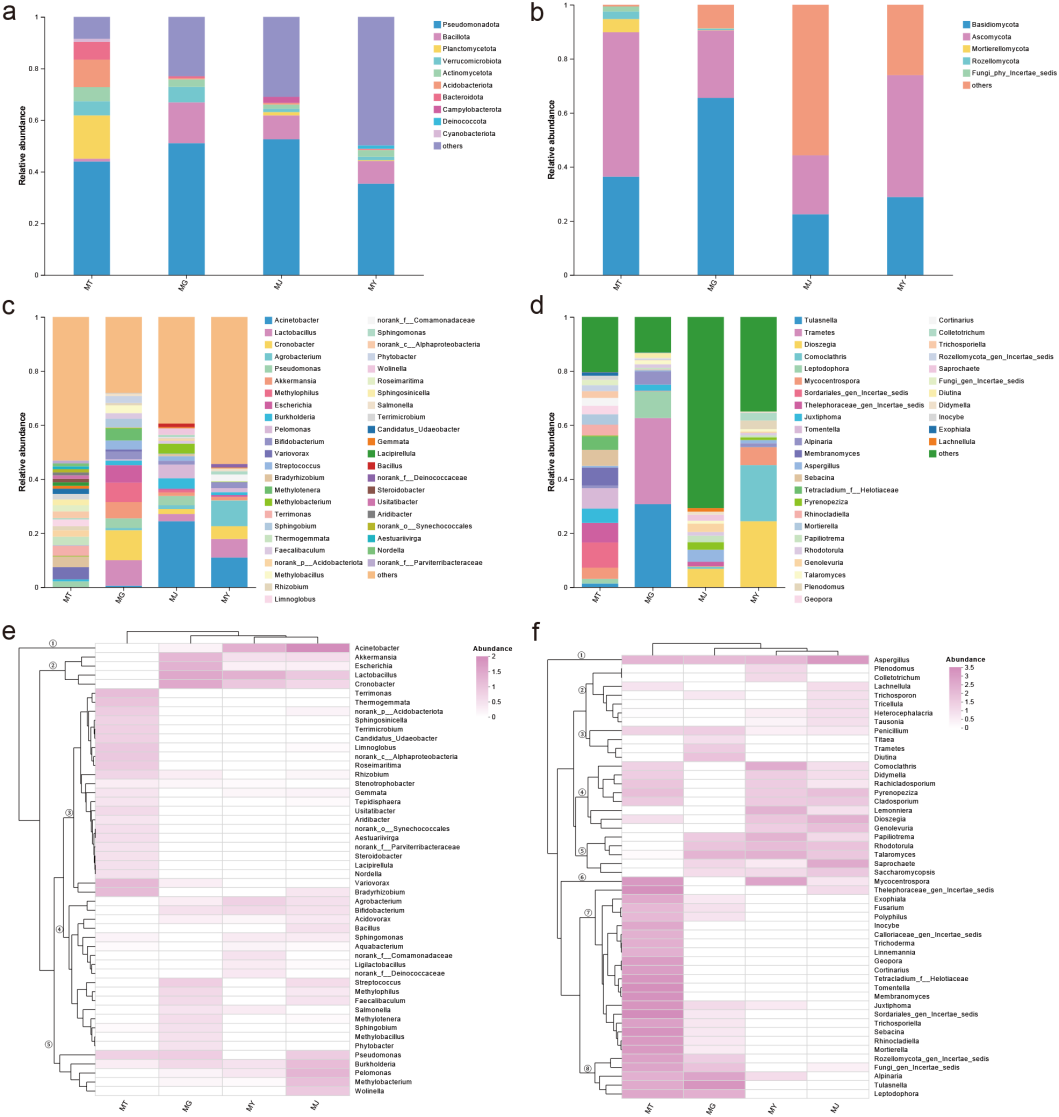
Composition and distribution of microbial communities across different compartments of *C. franchetii*: rhizosphere soil (MT) and endophytic tissues of the root (MG), stem (MJ), and leaf (MY). Bar plots show the relative abundance of bacterial **(A)** and fungal **(B)** communities at the phylum level, and bacterial **(C)** and fungal **(D)** communities at the genus level. Heatmaps display the top 50 bacterial genera **(E)** and the overall bacterial community composition **(F)** at the genus level.

At the phylum level, the top ten bacterial phyla in rhizosphere soil, roots, stems, and leaves were: Pseudomonadota (35.38–52.58%), Bacillota (1.08–15.81%), Planctomycetota (0.32–16.77%), Verrucomicrobiota (1.29–6.02%), Actinomycetota (1.61–5.48%), Acidobacteriota (0.22–10.65%), Bacteroidota (0.32–6.88%), Campylobacterota (2.37%), Deinococcota (1.29%), and Cyanobacteriota (1.18%). Among these, Bacteroidota was primarily distributed in rhizosphere soil and roots, Campylobacterota mainly in stems, Deinococcota predominantly in leaves, and Cyanobacteriota chiefly in rhizosphere soil. The top three bacterial phyla in rhizosphere soil were Pseudomonadota (43.98%), Planctomycetota (16.77%), and Acidobacteriota (10.65%). For endophytic bacteria in roots, stems, and leaves, the top two phyla were Pseudomonadota (35.38–51.08%) and Bacillota (8.82–15.81%). The third most abundant phylum in roots was Verrucomicrobiota (6.02%), in stems was Campylobacterota (2.37%), and in leaves was Actinomycetota (2.58%) (**Figure 4A**).

At the genus level, the top ten bacterial genera in rhizosphere soil, roots, stems, and leaves were: *Acinetobacter* (0.54–24.41%), *Lactobacillus* (2.69–9.46%), *Cronobacter* (1.83–11.08%), *Agrobacterium* (0.86–9.46%), *Pseudomonas* (0.11–3.55%), *Akkermansia* (1.29–6.02%), *Methylophilus* (0.86–7.20%), *Escherichia* (0.54–6.45%), *Burkholderia* (0.75–3.87%), and *Pelomonas* (0.43–5.05%). With the exception of Pseudomonas, which was predominantly distributed in rhizosphere soil, roots, and stems, *Methylophilus* primarily in roots and stems, and *Burkholderia* across all groups, the remaining genera were distributed in roots, stems, and leaves. The top three bacterial genera in rhizosphere soil were *Variovorax* (4.52%), *Bradyrhizobium* (3.98%), and *Terrimonas* (3.76%). In roots, the top three genera were *Cronobacter* (11.08%), *Lactobacillus* (9.46%), and *Methylophilus* (7.20%). In stems, the top three genera were *Acinetobacter* (24.41%), *Pelomonas* (5.05%), and *Burkholderia* (3.87%). In leaves, the top three genera were *Acinetobacter* (10.97%), *Agrobacterium* (9.46%), and *Lactobacillus* (6.88%) (**Figure 4C**).

At the phylum level, the top five fungal phyla in rhizosphere soil, roots, stems, and leaves were: Basidiomycota (22.47–66.54%), Ascomycota (21.80–53.45%), Mortierellomycota (0.09–4.88%), Rozellomycota (0.41–2.79%), and Fungi_phy_Incertae_sedis (0.03–1.86%). Among these, Basidiomycota and Ascomycota were predominantly distributed in rhizosphere soil as well as roots, stems, and leaves (**Figure 4B**).

At the genus level, the top ten fungal genera in rhizosphere soil, roots, stems, and leaves were: *Tulasnella* (1.27–30.72%), *Trametes* (31.88%), *Dioszegia* (6.80–24.41%), *Comoclathris* (0.79–20.78%), *Leptodophora* (1.67–9.96%), *Mycocentrospora* (0.23–6.62%), Sordariales_gen_Incertae_sedis (0.15–9.36%), Thelephoraceae_gen_Incertae_sedis (1.58–7.23%), *Juxtiphoma* (2.25–5.29%), and *Tomentella* (7.56%). Among these, *Tulasnella*, *Leptodophora*, *Juxtiphoma*, and Sordariales_gen_Incertae_sedis were predominantly distributed in rhizosphere soil and roots; *Dioszegia* and *Comoclathris* primarily in stems and leaves; Thelephoraceae_gen_Incertae_sedis chiefly in rhizosphere soil and stems; *Mycocentrospora* mainly in rhizosphere soil and leaves; while *Trametes* and *Tomentella* were concentrated primarily in rhizosphere soil. The top three fungal genera in rhizosphere soil were Sordariales_gen_Incertae_sedis (9.36%), *Tomentella* (7.56%), and Thelephoraceae_gen_Incertae_sedis (7.23%). In roots, the top three genera were *Trametes* (31.88%), *Tulasnella* (30.72%), and *Leptodophora* (9.96%). In stems, the top three genera were *Dioszegia* (6.80%), *Aspergillus* (4.46%), and *Genolevuria* (3.08%). In leaves, the top three genera were *Dioszegia* (24.41%), *Comoclathris* (20.78%), and *Mycocentrospora* (6.62%) (**Figure 4D**).

At the genus level, the top 50 bacterial genera were subjected to hierarchical clustering analysis and grouped into five clades. Clades 1, 2, and 4 exhibited higher relative abundances in roots, stems, and leaves, while clade 3 predominated in rhizosphere soil, and clade 5 showed elevated abundance in stems and leaves. Distinct microbial profiles were observed across rhizosphere soil, roots, stems, and leaves, with dominant genera varying among sample types. *Variovorax*, *Bradyrhizobium*, and *Terrimonas* were significantly more abundant in rhizosphere soil compared to plant tissues. *Cronobacter*, *Lactobacillus*, *Escherichia*, and *Akkermansia* were enriched in roots; *Acinetobacter*, *Pelomonas*, *Burkholderia*, and *Methylobacterium* were more prevalent in stems. In leaves, *Acinetobacter* abundance exceeded that in rhizosphere soil and roots but was lower than in stems, while *Lactobacillus* abundance surpassed levels in rhizosphere soil and stems but remained below root levels (**Figure 4E**).

For fungi, the top 50 genera formed eight clades. Clade 1 maintained high abundance across all sample types. Clade 2 predominated in stems, followed by leaves; clade 3 was enriched in roots; clades 4 and 6 showed higher abundance in rhizosphere soil, stems, and leaves but were nearly absent in roots; clade 5 was prominent in roots, stems, and leaves; clade 7 peaked in rhizosphere soil, then roots; clade 8 was elevated in rhizosphere soil and roots. Approximately half of fungal genera (e.g., Sordariales_gen_Incertae_sedis, *Tomentella*, Thelephoraceae_gen_Incertae_sedis) demonstrated significantly higher abundance in rhizosphere soil versus plant tissues. In roots, *Tulasnella*, *Alpinaria*, *Leptodophora*, and *Talaromyces* were prominent, with *Tulasnella*, *Alpinaria*, and *Leptodophora* abundance comparable to rhizosphere soil, while *Talaromyces* aligned with stem levels. *Aspergillus*, *Saprochaete*, and *Dioszegia* were enriched in stems; *Comoclathris*, *Talaromyces*, *Lemonniera*, and *Papiliotrema* dominated leaves, though *Mycocentrospora*—while highest in leaves—remained lower than in rhizosphere soil (**Figure 4F**).

Collectively, microbial communities in rhizosphere soil diverged substantially from those in plant tissues, with stems and leaves exhibiting higher intergroup similarity.

### 3.5 Functional gene potential prediction

**Figure 5:**
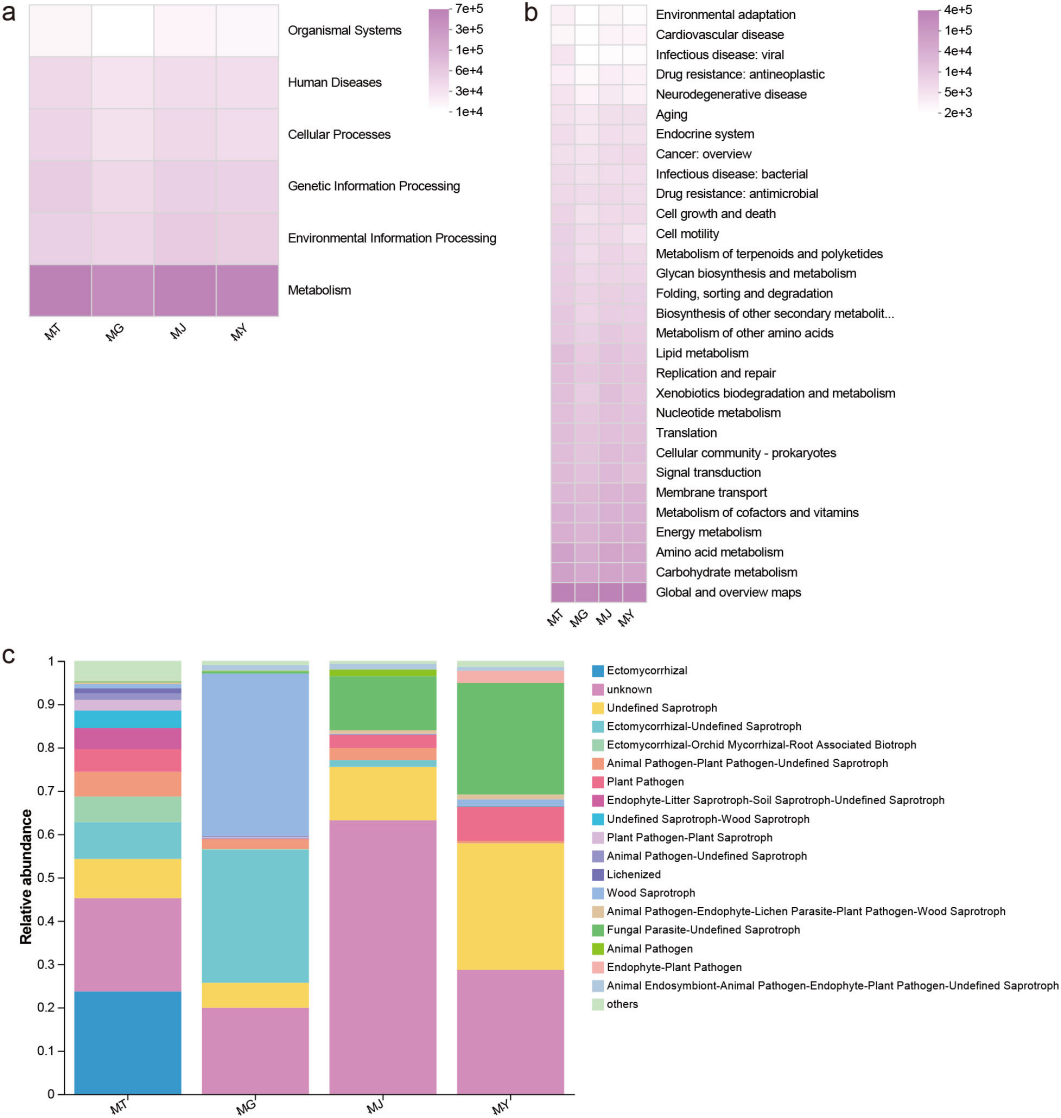
Functional annotation of microbial communities from different compartments of *C. franchetii*: rhizosphere soil (MT) and endophytic tissues of the root (MG), stem (MJ), and leaf (MY). Shown are the predicted bacterial functions inferred by PICRUSt at level 1 **(A)** and level 2 **(B)**, and the fungal functional guilds annotated by FunGuild **(C)**.

KEGG Level 1 functional annotations for bacteria in rhizosphere soil, root, stem, and leaf samples comprised Metabolism (75.23–77.00%), Environmental Information Processing (5.72–7.00%), Genetic Information Processing (5.94–6.50%), Cellular Processes (4.47–4.91%), Human Diseases (4.27–4.43%), and Organismal Systems (1.90–2.17%), with Metabolism representing the predominant functional pathway. At KEGG Level 2, among the top 30 functional pathways, 12 were metabolism-related; moreover, the top five pathways by relative abundance were all associated with metabolism (**Figures 5A, B**).

Fungal nutritional modes annotated in rhizosphere soil, root, stem, and leaf samples comprised 17 categories: Ectomycorrhizal, Undefined Saprotroph, Ectomycorrhizal-Undefined Saprotroph, Ectomycorrhizal-Orchid Mycorrhizal-Root Associated Biotroph, Animal Pathogen-Plant Pathogen-Undefined Saprotroph, Plant Pathogen, Endophyte-Litter Saprotroph-Soil Saprotroph-Undefined Saprotroph, Undefined Saprotroph-Wood Saprotroph, Plant Pathogen-Plant Saprotroph, Animal Pathogen-Undefined Saprotroph, Lichenized, Wood Saprotroph, Animal Pathogen-Endophyte-Lichen Parasite-Plant Pathogen-Wood Saprotroph, Fungal Parasite-Undefined Saprotroph, Animal Pathogen, Animal Endosymbiont-Animal Pathogen-Endophyte-Plant Pathogen-Undefined Saprotroph, and Endophyte-Plant Pathogen. Among these, Ectomycorrhizal and Undefined Saprotroph predominated in rhizosphere soil fungi, Wood Saprotroph and Ectomycorrhizal-Undefined Saprotroph were primary in root fungi, while Undefined Saprotroph and Fungal Parasite-Undefined Saprotroph were dominant in stem and leaf fungi (**Figure 5C**).

### 3.6 Differential species identification

**Figure 6:**
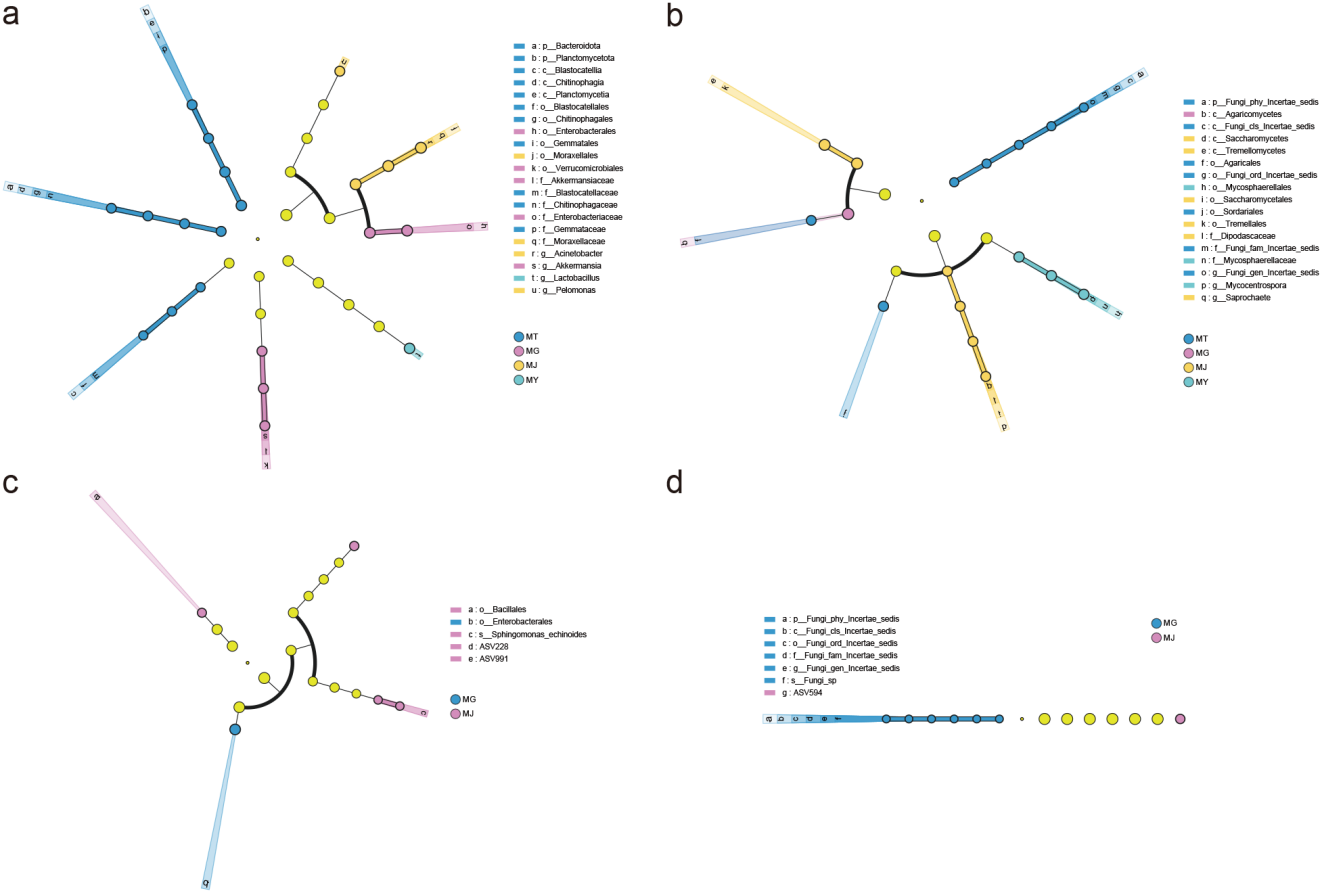
LEfSe analysis identifying differentially abundant bacteria **(A)** and fungi **(B)** between the rhizosphere soil (MT) and the endophytic tissues (root (MG), stem (MJ), and leaf (MY)); and differentially abundant bacteria **(C)** and fungi **(D)** among the root (MG), stem (MJ), and leaf (MY) endophytic tissues of *C. franchetii*.

LEfSe linear discriminant analysis identified 21 bacterial biomarker taxa through multi-level differential testing from phylum to genus (LDA>2, P<0.05), with distinct biomarker bacterial groups specific to rhizosphere soil, root, stem, and leaf samples. Rhizosphere soil contained 10 bacterial biomarkers, primarily including Bacteroidota, Planctomycetota, and Blastocatellia; roots featured 5 biomarkers, dominated by Enterobacterales and Verrucomicrobiales; stems harbored 4 biomarkers, principally Moraxellales and *Pelomonas*; while leaves exhibited one biomarker, *Lactobacillus* (**Figure 6A**).

Similarly, distinct fungal biomarker groups were identified across sample types, totaling 17 fungal biomarker taxa. Rhizosphere soil contained 7 biomarkers, chiefly Fungi_phy_Incertae_sedis, Agaricales, and Sordariales; roots featured one biomarker, Agaricomycetes; stems hosted 6 biomarkers, primarily Tremellomycetes and Saccharomycetes; and leaves presented 3 biomarkers, dominated by Mycosphaerellales (**Figure 6B**).

LEfSe analysis restricted to plant tissue samples identified differentially enriched bacteria only in roots and stems through multi-level differential testing from phylum to ASV (LDA>2, P<0.05), revealing five bacterial biomarker taxa. Roots contained one biomarker, Enterobacterales, while stems featured four biomarkers, predominantly Bacillales and *Sphingomonas_echinoides* (**Figure 6C**). Similarly, differentially enriched fungi were identified in roots and stems, totaling seven fungal biomarker taxa. Roots harbored six biomarkers, primarily including Fungi_phy_Incertae_sedis; stems exhibited one biomarker, ASV594 (**Figure 6D**).

### 3.7 Integrated analysis of metabolic patterns, differential metabolites, and enriched pathways in plant organs

**Figure 7:**
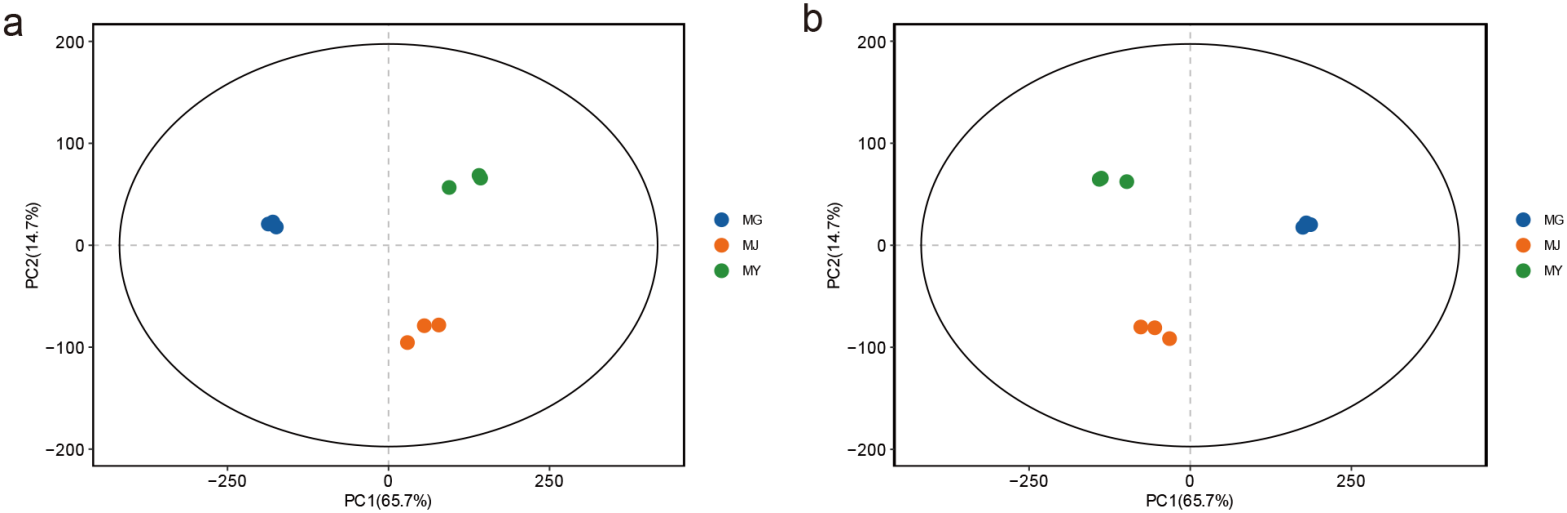
Principal component analysis (PCA) **(A)** and partial least squares-discriminant analysis (PLS-DA) **(B)** of metabolites from different endophytic tissues of *C. franchetii*: root (MG), stem (MJ), and leaf (MY).

**Figure 8:**
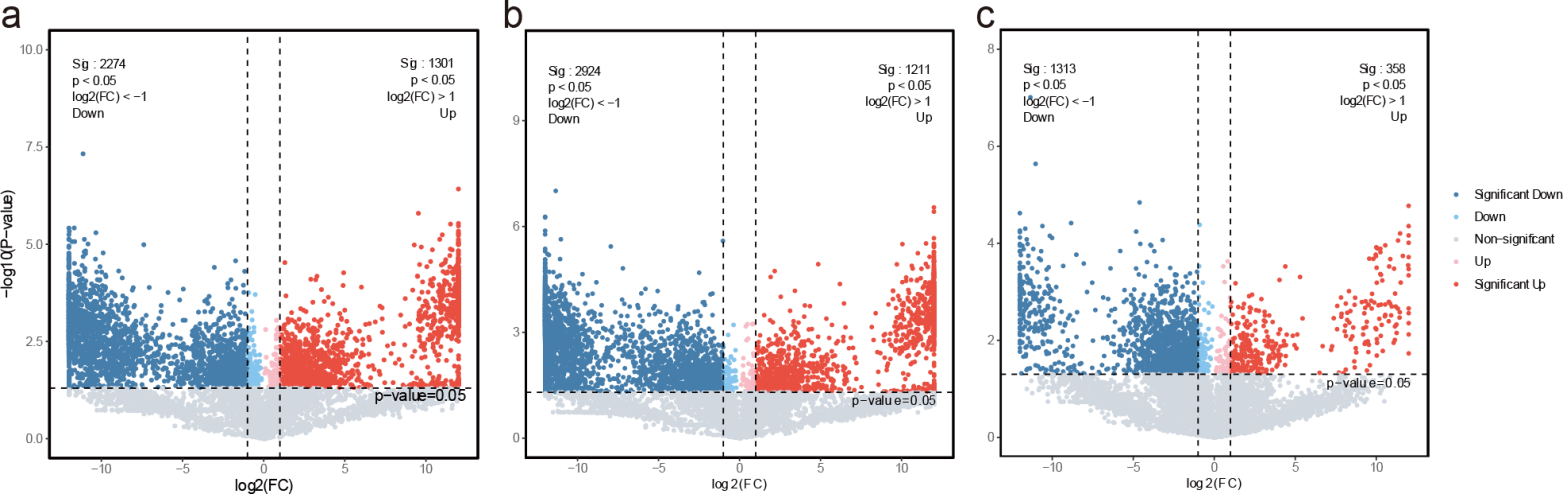
Volcano plots of significantly differential metabolites from pairwise comparisons across the endophytic tissues of *C. franchetii*: root vs. stem **(A)**, root vs. leaf **(B)**, and stem vs. leaf **(C)**.

Metabolic profile pattern recognition analysis via PCA and PLS-DA revealed that PC1 (65.7%) and PC2 (14.7%) collectively explained 80.4% of the variation, with distinct intergroup sample differentiation and robust intragroup clustering observed, indicating the reliability of data processing for the three sample groups (**Figures 7A, B**). Based on this reliable data processing, significant differential metabolites were screened among the three comparison groups using the criteria of p-value < 0.05 and FC ≥ 2.0 or FC ≤ 1/2.0, where the root-stem comparison identified 3,575 differential metabolites comprising 1,301 upregulated and 2,274 downregulated metabolites (**Figure 8A**), the root-leaf comparison yielded 4,135 differential metabolites with 1,211 upregulated and 2,924 downregulated (**Figure 8B**), and the stem-leaf comparison detected 1,671 differential metabolites including 358 upregulated and 1,313 downregulated metabolites (**Figure 8C**).

**Figure 9:**
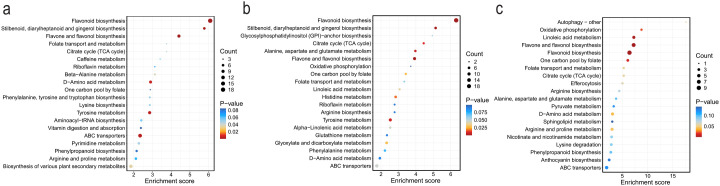
Top 20 most significantly enriched KEGG pathways identified from pairwise comparisons across the endophytic tissues of *C. franchetii*: root vs. stem **(A)**, root vs. leaf **(B)**, and stem vs. leaf **(C)**.

Thirteen metabolic pathways showed significant differences between roots and stems (P < 0.05), including Flavonoid biosynthesis, Flavone and flavonol biosynthesis, Stilbenoid, diarylheptanoid and gingerol biosynthesis, ABC transporters, Tyrosine metabolism, and Citrate cycle (TCA cycle) (**Figure 9A**). Twelve metabolic pathways exhibited significant differences between roots and leaves (P < 0.05), such as Flavonoid biosynthesis, Flavone and flavonol biosynthesis, Stilbenoid, diarylheptanoid and gingerol biosynthesis, Tyrosine metabolism, Citrate cycle (TCA cycle), One carbon pool by folate, Linoleic acid metabolism, and ABC transporters (**Figure 9B**). Seven metabolic pathways demonstrated significant differences between stems and leaves (P < 0.05), including Flavonoid biosynthesis, Flavone and flavonol biosynthesis, Linoleic acid metabolism, and One carbon pool by folate (**Figure 9C**).

Flavonoid biosynthesis and Flavone and flavonol biosynthesis pathways exhibited significant differential expression across all three comparison groups, suggesting their potential role as key metabolic pathways governing differentiation among roots, stems, and leaves, with C. franchetii potentially regulating these pathways to adapt to functional demands of distinct organs; Stilbenoid, diarylheptanoid and gingerol biosynthesis, ABC transporters, Tyrosine metabolism, and Citrate cycle (TCA cycle) were consistently differentiated between roots versus stems and roots versus leaves, implying unique functions of roots in secondary defense metabolism, substance transport, energy metabolism, and amino acid metabolism; Linoleic acid metabolism and One carbon pool by folate (a central metabolic network where folate derivatives carry and transfer one-carbon units for nucleotide synthesis and methylation reactions) showed significant alterations in both leaf-root and leaf-stem comparisons, indicating specialized roles of leaves in lipid metabolism and one-carbon unit metabolism; and D-Amino acid metabolism was differentially expressed in stem-root and stem-leaf comparisons, potentially implicating stems in transitional or regulatory functions within D-amino acid metabolism.

### 3.8 Correlation profiling of ASV-resolved differential microbiota and top p-value ranked metabolites

**Figure 10:**
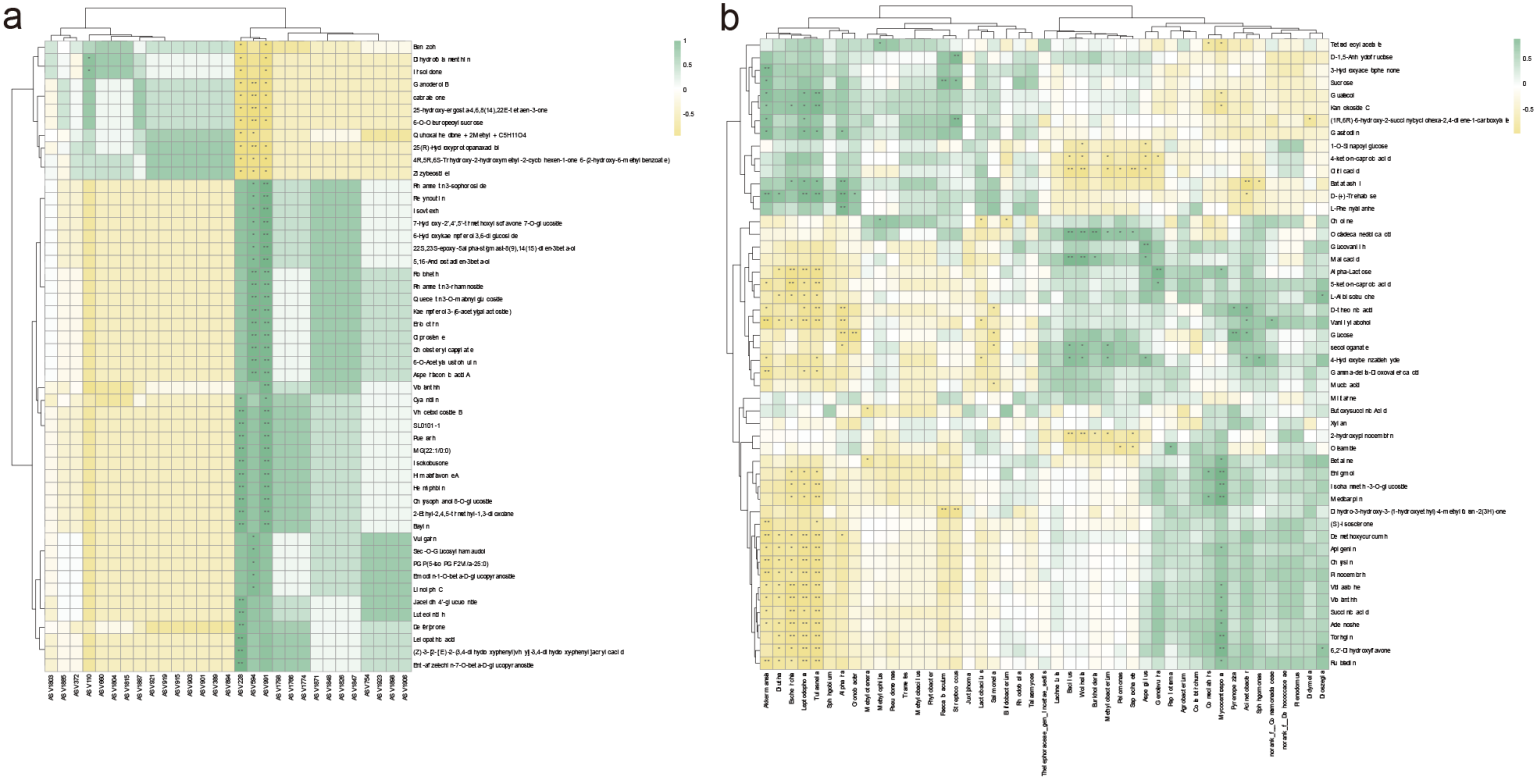
Correlation of root-stem differential microbes and root-stem-leaf core microbiota with metabolites. Correlation heatmap of Root-Stem Differential ASVs vs. Top 50 Differential KEGG L1 Metabolites **(A)**. Correlation heatmap of Core Genera (relative abundance ≥ 1% in root, stem, or leaf) vs. Top 50 Abundant KEGG L1 Metabolites **(B)**.

Correlation analysis between differential microorganisms (23 bacterial ASVs and 6 fungal ASVs) at the ASV level in roots and stems, and the top 50 significantly differential metabolites at level 1 revealed significant associations. Bacterial ASV228 exhibited significant positive correlations with 17 metabolites, including Leiopathic acid, (Z)-3-[2-[(E)-2-(3,4-dihydroxyphenyl)vinyl]-3,4-dihydroxy-phenyl]acrylic acid, and Ent-afzelechin-7-O-beta-D-glucopyranoside, while showing significant negative correlations with 11 metabolites such as Benzoin, Dihydrofoliamenthin, and Irisolidone. Fungal ASV594 demonstrated significant positive correlations with 21 metabolites, including Cholesteryl caprylate, 6-O-Acetylaustroinulin, and Asperitaconic acid A, and significant negative correlations with 8 metabolites including Ganoderol B, cabraleone, and 25-hydroxy-ergosta-4,6,8(14),22E-tetraen-3-one. Bacterial ASV991 showed significant positive correlations with 28 metabolites such as Rhamnetin 3-sophoroside, Reynoutrin, and Isovitexin, and significant negative correlations with 10 metabolites including Benzoin, Dihydrofoliamenthin, and Irisolidone. Fungal ASV110 displayed significant positive correlations with Dihydrofoliamenthin and Irisolidone (**Figure 10A**).

### 3.9 Integrated correlation profiling of abundance-weighted genera and top-ranked metabolites across plant compartments

Correlation analysis between microbial genera with relative abundance ≥1% in roots, stems, and leaves and the top 50 metabolites by abundance at level 1 revealed significant associations. Among 45 microbial genera, 31 exhibited significant correlations with 48 metabolites. Notably, *Akkermansia*, *Diutina*, *Escherichia*, *Leptodophora*, and *Tulasnella* demonstrated extensive positive and negative correlations with multiple metabolites(**Figure 10b**).

Specifically, *Akkermansia* showed significant positive correlations with seven metabolites (3-Hydroxyacetophenone, D-(+)-Trehalose, Sucrose) and significant negative correlations with fifteen metabolites (Vanillyl alcohol, Gamma-delta-Dioxovaleric acid, (S)-Isosclerone). *Diutina* exhibited significant positive correlation with D-(+)-Trehalose and significant negative correlations with thirteen metabolites (Toringin, 6,2’-Dihydroxyflavone, Rubiadin). *Escherichia* demonstrated significant positive correlations with Kanokoside C and Batatasin I, and significant negative correlations with eighteen metabolites (5-keto-n-caproic acid, Alpha-Lactose, Vidarabine). *Leptodophora* displayed significant positive correlations with six metabolites (D-(+)-Trehalose, Batatasin I, Gastrodin) and significant negative correlations with twenty metabolites (Toringin, 6,2’-Dihydroxyflavone, Rubiadin). *Tulasnella* exhibited significant positive correlations with five metabolites (Guaiacol, Kanokoside C, D-(+)-Trehalose) and significant negative correlations with twenty-two metabolites (Toringin, 6,2’-Dihydroxyflavone, Rubiadin).

*Alpinaria* exhibited significant positive correlations with Batatasin I, D-(+)-Trehalose, L-Phenylalanine, and Gastrodin, while showing significant negative correlations with five metabolites including D-threonic acid, Vanillyl alcohol, and Glucose. *Cronobacter* demonstrated significant positive correlation with D-(+)-Trehalose and significant negative correlation with Glucose. *Methylotenera* displayed significant negative correlations with Butoxysuccinic Acid and Betaine. *Methylophilus* showed significant positive correlations with Tetradecyl acetate and Choline. *Faecalibaculum* exhibited significant positive correlation with Sucrose and significant negative correlation with Dihydro-3-hydroxy-3-(1-hydroxyethyl)-4-methylfuran-2(3H)-one. *Streptococcus* demonstrated significant positive correlations with D-1,5-Anhydrofructose, Sucrose, and (1R,6R)-6-hydroxy-2-succinylcyclohexa-2,4-diene-1-carboxylate, and significant negative correlation with Dihydro-3-hydroxy-3-(1-hydroxyethyl)-4-methylfuran-2(3H)-one. *Lactobacillus* showed significant negative correlations with Choline, Vanillyl alcohol, and 4-Hydroxybenzaldehyde. *Salmonella* exhibited significant negative correlations with D-threonic acid, Glucose, secologanate, and Mucic acid. Bifidobacterium displayed significant negative correlation with Choline. *Bacillus* and *Wolinella* showed significant positive correlations with Octadecanedioic acid, Malic acid, secologanate, and 4-Hydroxybenzaldehyde, and significant negative correlations with 4-keto-n-caproic acid, Citric acid, and 2-hydroxypinocembrin; additionally, *Wolinella* demonstrated significant negative correlation with 1-O-Sinapoylglucose. *Burkholderia* exhibited significant positive correlations with Octadecanedioic acid and Malic acid, and significant negative correlation with 2-hydroxypinocembrin. *Methylobacterium* exhibited significant positive correlations with Octadecanedioic acid, secologanate, and 4-Hydroxybenzaldehyde, and significant negative correlations with 4-keto-n-caproic acid, Citric acid, and 2-hydroxypinocembrin. *Pelomonas* showed significant positive correlation with Octadecanedioic acid and significant negative correlations with Citric acid and Oleamide. *Saprochaete* demonstrated significant positive correlation with Octadecanedioic acid and significant negative correlations with Citric acid, 2-hydroxypinocembrin, and Oleamide. *Aspergillus* exhibited significant positive correlations with Glucovanillin, Malic acid, and 4-Hydroxybenzaldehyde, and significant negative correlations with 1-O-Sinapoylglucose, 4-keto-n-caproic acid, and Citric acid. *Genolevuria* showed significant positive correlations with Alpha-Lactose and 5-keto-n-caproic acid, and significant negative correlation with 4-keto-n-caproic acid. *Papiliotrema* displayed significant positive correlation with Oleamide. *Comoclathris* exhibited significant positive correlations with Enigmol and Medicarpin, and significant negative correlation with Tetradecyl acetate. *Mycocentrospora* showed significant positive correlations with 13 metabolites including Enigmol, Isorhamnetin-3-O-glucoside, and Medicarpin, and significant negative correlations with Tetradecyl acetate, Guaiacol, and Kanokoside C. *Pyrenopeziza* demonstrated significant positive correlations with Glucose and D-threonic acid. *Acinetobacter* exhibited significant positive correlations with D-threonic acid, Vanillyl alcohol, Glucose, and 4-Hydroxybenzaldehyde, and significant negative correlations with Batatasin I and D-(+)-Trehalose. *Sphingomonas* showed significant positive correlation with 4-Hydroxybenzaldehyde and significant negative correlation with Batatasin I. norank_f:Comamonadaceae displayed significant positive correlation with Vanillyl alcohol. *Didymella* exhibited significant negative correlation with (1R,6R)-6-hydroxy-2-succinylcyclohexa-2,4-diene-1-carboxylate. *Dioszegia* demonstrated significant positive correlations with L-Alloisoleucine and 6,2’-Dihydroxyflavone.

## 4 Discussion

### 4.1 Core microbial conservation vs. habitat specificity: structure-function analysis of symbiotic communities in *Cypripedium* and implications for conservation

The characterization of symbiotic microbial communities in *Cypripedium* species is crucial for understanding their ecological adaptability and formulating effective conservation strategies. Extensive research indicates that their symbiotic fungal communities are predominantly composed of Basidiomycota and Ascomycota. Among these, Tulasnellaceae and *Tulasnella* exhibit significant dominance or high frequency across multiple species and geographical regions, highlighting their widespread and conserved association with *Cypripedium* mycorrhizal symbiosis. This pattern has been consistently observed in *C. tibeticum*, *C. flavum*, and *C. bardolphianum* from Huanglong Valley, Sichuan (Xu et al., 2019); *C. flavum* from Northwest Yunnan (Miao et al., 2015); and 44 *Cypripedium* populations across Europe and North America (Shefferson et al., 2005). Additionally, *Tulasnella* demonstrates host specificity in C. flavum from Yunnan (Quan et al., 2015) and *C. plectrochilum* from Northwest Yunnan (An, 2023), though its composition is also influenced by habitat. For instance, *C. macranthos* from Mount Wutai, Shanxi, is dominated by Tulasnellaceae and Epulorhiza (Fan et al., 2023), while *C. macranthum* from Baihua Mountain, Beijing, exhibits Ascomycota, Zygomycota, and Basidiomycota as dominant fungal phyla (Fu et al., 2019; Zhang et al., 2017). Significant geographical and interspecific variations exist: *C. calceolus* in the Changbai Mountains is associated with *Cadophora* as the dominant endophytic fungus (Shan et al., 2024), whereas *C. calceolus* in Northeast China coexists with multiple taxa, including Tulasnellaceae, Psathyrellaceae, and Herpotrichiellaceae (Liu et al., 2023a).

Analysis of *C. franchetii* in this study revealed Basidiomycota and Ascomycota as dominant fungal phyla, aligning with findings from Changbai Mountains (Shan et al., 2024), Shanxi (Zhou et al., 2024a), Heilongjiang (Qian et al., 2025), and Jinfo Mountain (Qiao, 2011). The dominance of *Tulasnella* at the genus level strongly supports its universal and critical role in *Cypripedium* (Xu et al., 2019; Shefferson et al., 2005; Miao et al., 2015; An, 2024). However, this study also identified *Trametes*, *Dioszegia*, and *Comoclathris* as tissue-dominant fungi, contrasting with *Trichoderma* dominance in *C. japonicum* from Mount Deogyu, Korea (Gang et al., 2017); *Cadophora* dominance in *C. calceolus* from Changbai Mountains (Shan et al., 2024); and high Sebacinaceae and Serendipitaceae abundance in the rhizosphere of *C. japonicum* from Tianmu Mountain (Zhou, 2020). These differences corroborate the observation that “symbiotic fungal communities in Cypripedium vary significantly across habitats” (Zhang, 2013).

Regarding bacterial communities, Pseudomonadota (formerly Proteobacteria) dominated the rhizosphere soil in this study. This aligns with the high abundance of Pseudomonadaceae in endophytic bacteria of *Cypripedium* from Changbai Mountains (Shan et al., 2024) and the dominance of Proteobacteria in Baihua Mountain studies (Fu et al., 2019; Zhang et al., 2017), suggesting this phylum is ubiquitous in *Cypripedium* rhizosphere and endophytic environments. However, dominant genera identified here (*Acinetobacter*, *Lactobacillus*, *Cronobacter*) differ from previously reported taxa, revealing species- or habitat-specific bacterial communities at finer taxonomic levels.

In summary, the symbiotic microbial communities of *Cypripedium* species exhibit a dual pattern of “core conservation” and “environmental shaping.” Fungal communities reveal a conserved symbiotic core across species and geographical regions, dominated by Basidiomycota and Ascomycota, particularly the genus *Tulasnella*. However, specific dominant genera vary considerably depending on host species, organ type, and habitat. Bacterial communities show certain commonalities at the phylum level (e.g., the widespread importance of Pseudomonadota), but exhibit greater variability at the genus level. These findings suggest that in future *ex situ* conservation and reintroduction practices, beyond the widely emphasized *Tulasnella*-like fungi, it is essential to comprehensively consider the functional roles of key bacteria and characteristic fungal groups (e.g., *Trametes* and *Dioszegia* identified in this study) in specific habitats, as well as the potential contributions of key fungi and their accessory microorganisms to successful cultivation, as indicated by Cho et al. (2022). This integrated approach will enable the development of more targeted microbial community management strategies.

### 4.2 Tissue specificity and niche differentiation: gradational distribution and functional adaptation of microbial communities from the rhizosphere to the endosphere of *C. franchetii*

This study reveals significant differences in the composition, diversity, and functionality of microbial communities between the rhizosphere soil and plant tissues (root, stem, leaf) of *C. franchetii*. The bacterial and fungal communities in the rhizosphere soil exhibited the highest richness and diversity, whereas the internal plant tissues showed lower diversity. Specifically, bacterial diversity in leaves was significantly lower than in stems, and root fungal richness was the lowest. This pattern reflects strong niche selection pressure. The rhizosphere soil serves as a “hotspot” for plant-microbe interactions, characterized by abundant resources and a relatively open environment that supports higher microbial diversity (Meeds et al., 2019). The collective genome of rhizosphere microbial communities is termed the “second genome” of plants, with soil microbes acting as the most crucial “seed bank” (Berendsen et al., 2012; Bulgarelli et al., 2013). The high α-diversity in the rhizosphere is attributed to substantial resource heterogeneity and broad ecological niches (Chu et al., 2020). Conversely, the lower α-diversity within plant tissues results primarily from host selective filtering, where plants rigorously regulate colonizing microbial taxa through immune systems and physicochemical barriers (e.g., UV radiation on leaf surfaces, desiccation, and antimicrobial compounds) (Yuan et al., 2008).

β-Diversity analysis indicated highly significant differences in microbial community structure between rhizosphere soil and plant tissues. Notably, bacterial communities in roots and leaves showed greater structural similarity compared to their respective differences with stems. This suggests that despite distinct habitats, roots and leaves—as critical interfaces for plant-environment exchange—may experience similar selection pressures. Fungal communities, however, exhibited pronounced differences among roots, stems, and leaves, reflecting high specificity in adaptation to distinct tissue microenvironments (Guo et al., 2020).

Functionally, regardless of colonization site, bacterial communities consistently prioritized “metabolism” pathways, particularly those related to carbohydrate, amino acid, and energy metabolism. This forms the functional foundation for microbial survival and responsiveness in plant-associated habitats, aligning with the “rhizosphere life community” theory. This theory posits a coupled system where plant-driven carbon flow and microbe-mediated nutrient feedback interact (Zhang et al., 2025). Fungal nutritional modes displayed a gradient of ecological strategies: rhizosphere soil was dominated by ectomycorrhizal fungi and saprotrophs, specializing in organic matter decomposition and symbiotic nutrient mobilization (Meeds et al., 2021; Shi et al., 2022; Dai et al., 2025). Root fungi included symbiotic taxa alongside an increased proportion of xylem saprotrophs, indicative of a transitional niche. Stem and leaf fungal communities were predominantly saprotrophic or “mycoparasitic-saprotrophic,” suggesting ecological roles in tissue degradation, endophytism, or biocontrol via parasitism of other fungi (Feng et al., 2019; Liu et al., 2023b; Gao et al., 2021). This continuous shift in fungal nutritional strategies from rhizosphere to leaves provides direct evidence of host selection and niche differentiation. It demonstrates that distinct plant tissues shape functionally specialized microbial communities by offering unique physicochemical microenvironments and carbon sources (Martínez-Diz et al., 2019; Faticov et al., 2023).

### 4.3 LEfSe biomarker identification in plant microbiome studies: a critical examination of statistical associations versus ecological causality

This study preliminarily revealed the specific distribution patterns of bacterial and fungal communities across the tissue gradient from rhizosphere soil to roots, stems, and leaves through LEfSe analysis, providing important preliminary clues and hypotheses for deciphering the spatial assembly patterns of plant microbiomes. This analysis, based on rigorous statistical comparisons, identified differential biomarkers indicating significantly enriched taxonomic units in distinct habitats, with clear statistical significance. For instance, the significant enrichment of *Lactobacillus* in leaves and Enterobacterales in roots aligns with recently reported patterns of microbial habitat specificity in plants (Zeng et al., 2023), enhancing the biological plausibility of our findings.

It should be noted that differential analysis methods such as LEfSe identify biomarkers as relatively enriched features within specific grouping frameworks. This study also observed that when the grouping strategy shifted from “all samples” (including soil) to “plant tissues only,” the number of root biomarkers changed, reflecting the context-dependent nature of biomarker identification. Furthermore, such methods are sensitive to sample size, data distribution, and other conditions, and are typically suited for inter-group comparisons, while exhibiting limitations when handling continuous environmental gradients (Segata et al., 2011). Consequently, although these differentially enriched biomarkers identified via amplicon sequencing and bioinformatic screening provide valuable correlative insights, their specific ecological functions and causal mechanisms require further rigorous experimental validation for definitive establishment.

### 4.4 Correlation analysis reveals the potential and limitations of plant-microbe metabolic interaction networks

This study preliminarily constructed a potential interaction network between specific microorganisms and plant metabolites through correlation analysis. Recent multi-omics studies have demonstrated that such associations represent significant manifestations of the complex plant-microbe-environment interaction system (Fan et al., 2025). At the amplicon sequence variant (ASV) level, extensive correlations were observed between differential microorganisms and differential metabolites. For instance, bacterial ASV228 showed significant positive correlations with 17 metabolites, including leiopathic acid. These highly specific associations suggest that particular microbial strains may directly participate in the synthesis or transformation of related metabolites. Recent research has confirmed the existence of numerous positive and negative associations in the co-occurrence networks between rhizosphere metabolites and microbial ASVs, where specific metabolites (e.g., serotonin and chlorogenic acid) act as network hubs, exhibiting significant correlations with multiple ASVs (Baker et al., 2024). Additionally, the association between fungal ASV594 and sterols (e.g., ganoderol B) aligns with the known functions of fungi in secondary metabolite synthesis (Wu et al., 2026). However, high-resolution ASV-level correlations primarily provide hypothetical clues for subsequent research, and their specific functional mechanisms require experimental validation.

At the microbial genus level, correlation analysis revealed a broader interaction network. Among 45 dominant genera, 31 genera exhibited significant correlations with 48 high-abundance metabolites. These association patterns can be partially explained by previously reported microbial metabolic functions: *Akkermansia* showed positive correlations with multiple carbohydrates and negative correlations with certain phenolic compounds. Research over the past five years has elucidated that *A. muciniphila* influences host metabolism and immunity through multiple pathways, including mucin degradation, short-chain fatty acid production, and modulation of bile acid and tryptophan metabolism (Lu, 2021; Xu et al., 2025; Liu et al., 2025), with its analogous metabolic interactions in the plant rhizosphere gaining increasing attention. *Escherichia* and *Lactobacillus* exhibit diverse metabolic functions and are known to participate in the synthesis and decomposition of various amino acids, carbohydrates, and secondary metabolites (Liu et al., 2024). Their positive and negative correlations with specific metabolites may reflect biosynthetic capabilities or nutrient competition relationships. Meanwhile, common plant growth-promoting rhizobacteria (PGPR) such as *Bacillus*, *Burkholderia*, and *Pseudomonas* have been shown in multiple recent studies to respond to root exudates and assist plants in resisting biotic and abiotic stresses by producing specific metabolites or inducing plant systemic resistance (Zhang et al., 2024; Zhou et al., 2024b; You et al., 2024; Li et al., 2024b). For example, *Pseudomonas* can be recruited by specific plant flavonoids (Gu et al., 2024), which may directly correspond to the correlation patterns observed in this study.

It should be noted that the above interpretations integrate the reported general metabolic functions of microorganisms with the correlation patterns observed in this study. This research and similar correlation analyses face clear limitations: analyses typically employ univariate correlation coefficients (e.g., Spearman or Pearson) without controlling for potential confounding variables such as sample batch, plant genotype, and soil physicochemical properties, which may lead to spurious associations. Additionally, correlations alone cannot distinguish causality—whether microbial activity drives metabolite changes, metabolite environments shape microbial communities, or both are regulated by third factors such as plant physiological status. Recent studies have revealed that plant secondary metabolites (e.g., benzoxazinoids) can reshape the rhizosphere microbiome, indirectly influencing nematode behavior (Wu et al., 2026), highlighting the complexity of interaction directionality. In summary, microbe-metabolite correlation analysis serves as a powerful hypothesis-generating tool in multi-omics research, enabling precise identification of potential key microbial and metabolite targets. However, translating statistical associations into reliable biological conclusions requires rigorous experimental functional validation. Future research trends will focus on integrating multi-omics correlation analysis with experimental approaches such as synthetic microbial communities and exogenous metabolite supplementation to ultimately elucidate specific interaction mechanisms (Mhlongo et al., 2018).

## 5 Conclusion

This study specifically reveals the characteristics of tissue-specific microecological structures and metabolomic profiles in *C. franchetii* during the critical post-flowering nutrient accumulation phase, which is crucial for resource allocation and subsequent plant development. Our work systematically depicts, for the first time, the tissue-specific microbiome-metabolome association map during this key period, yielding the following principal conclusions: (1) Microbial community assembly exhibits a synergistic mechanism of “core-conserved” and “habitat-specific” elements. Fungal communities are dominated by Basidiomycota and Ascomycota, with the widespread presence of Tulasnella confirming its conservation in Cypripedium symbiotic systems. However, specific dominant taxa (e.g., Trametes in roots, Dioszegia and Comoclathris in stems/leaves) and bacterial genera (e.g., Lactobacillus, Acinetobacter) display strong tissue specificity, indicating fine-scale environmental filtering by localized microenvironments. (2) Rhizosphere soil and different plant tissues form gradient-differentiated micro-niches. Microbial diversity significantly decreases from rhizosphere soil toward internal plant tissues (roots → stems → leaves), reflecting increasing host selection pressure. Fungal functional groups undergo regular succession along this gradient: shifting from primarily symbiotic/saprophytic types in the rhizosphere to predominantly saprophytic/parasitic types in stems and leaves, demonstrating adaptive differentiation of microbial ecological strategies to tissue-specific microenvironments. (3) Correlation analysis reveals extensive and specific potential interaction networks between microbial taxa and host plant metabolites. This suggests that specific microorganisms may deeply participate in or regulate the host’s secondary metabolic processes. Our findings emphasize that effective conservation of *C. franchetii* requires consideration of both their conserved symbiotic core (e.g., Tulasnella) and habitat-specific key microbial taxa. Artificial propagation should aim to construct synergistic microbial communities with greater ecological integrity. Furthermore, the discovered “microbe-metabolite” associations provide a novel theoretical basis and research leads for future targeted manipulation of microbiomes to influence the synthesis of bioactive compounds in medicinal plants.

## Data Availability

The datasets presented in this study can be found in online repositories. The names of the repository/repositories and accession number(s) can be found in the article/supplementary material.
